# Microorganisms—An Effective Tool to Intensify the Utilization of Sulforaphane

**DOI:** 10.3390/foods11233775

**Published:** 2022-11-23

**Authors:** Xiude Li, Yihan Wang, Guoping Zhao, Guangmin Liu, Pengjie Wang, Jinwang Li

**Affiliations:** 1 School of Food and Health, Beijing Technology and Business University, Beijing 100048, China; 2 Key Laboratory of Functional Dairy, Ministry of Education, Department of Nutrition and Health, China Agricultural University, No. 17 Qinghua East Road, Haidian District, Beijing 100083, China; 3 Institute of Agri-Food Processing and Nutrition, Beijing Academy of Agricultural and Forestry Sciences, Beijing 100097, China

**Keywords:** sulforaphane, microorganisms, myrosinase synthesis, gene modification, microencapsulation

## Abstract

Sulforaphane (SFN) was generated by the hydrolysis of glucoraphanin under the action of myrosinase. However, due to the instability of SFN, the bioavailability of SFN was limited. Meanwhile, the gut flora obtained the ability to synthesize myrosinase and glucoraphanin, which could be converted into SFN in the intestine. However, the ability of microorganisms to synthesize myrosinase in the gut was limited. Therefore, microorganisms with myrosinase synthesis ability need to be supplemented. With the development of research, microorganisms with high levels of myrosinase synthesis could be obtained by artificial selection and gene modification. Researchers found the SFN production rate of the transformed microorganisms could be significantly improved. However, despite applying transformation technology and regulating nutrients to microorganisms, it still could not provide the best efficiency during generating SFN and could not accomplish colonization in the intestine. Due to the great effect of microencapsulation on improving the colonization ability of microorganisms, microencapsulation is currently an important way to deliver microorganisms into the gut. This article mainly analyzed the possibility of obtaining SFN-producing microorganisms through gene modification and delivering them to the gut via microencapsulation to improve the utilization rate of SFN. It could provide a theoretical basis for expanding the application scope of SFN.

## 1. Introduction

Sulforaphane (1-isothiocyanate-4-(methylsulfonyl) butane, SFN) is produced by the degradation of glucoraphanin under the action of myrosinase (β-D-thioglucosidase) [1,2,3]. SFN and its precursor (glucoraphanin) are important safeguard substances used by plants to defend themselves against insects, pathogens, and herbivores [4]. Recently, researchers have found that SFN possesses antioxidant, anti-inflammatory, and antibacterial functions [5,6]. In addition, SFN could prevent and cure cancer [7,8,9], improve the nervous system [10,11,12], and prevent coronavirus disease 2019 [13]. Therefore, SFN has attracted extensive attention from researchers around the world.

Although SFN has shown great positive effects on human health, it is an unstable compound. Therefore, it is difficult to obtain SFN directly from cruciferous plant tissues owing to its structural instability [14,15,16,17]. It has always been a research topic of interest to maintain the function of SFN stably in the human body. Exogenous hydrolysis and transformation research of glucoraphanin have suggested that SFN could be obtained outside plant tissues with high biological activity. Meanwhile, the key controlling factors for the exogenous transformation are glucoraphanin concentration and the activity of myrosinase [18,19]. Triska et al. found that SFN formation was controlled by a temperature-specific epithiospecifier protein (a myrosinase cofactor). The most suitable exogenous transformation condition is to ensure sufficient radish sprout content to continuously add myrosinase and to maintain a stable transformation temperature during the process [20].

However, external factors can also affect myrosinase synthesis during the cultivation of cruciferous plants. Polyethylene glycol can enhance the expression of the myrosinase gene, which can increase myrosinase synthesis [21]. Lower planting temperatures can also result in higher activity of the myrosinase obtained from cruciferous plants and increase the ability of myrosinase to convert glucoraphanin into SFN [22]. Although the production of plant-derived SFN is increased through exogenous environmental regulation, it is difficult for the human body to take up enough SFN from plant tissues due to the instability of the SFN structure [23]. Therefore, determining how to enhance the absorption rate of SFN in intestinal tissues has attracted the attention of researchers.

Studies show that the gut microbiome not only helps digest food ingested into the gastrointestinal tract, but also converts dietary pairs into more active products [24,25,26]. Lai et al. showed that after feeding F344 rats with cooked *broccoli* (without myrosinase), the microbes in the gut of the rats converted glucoraphanin into SFN, and SFN was detected in the blood [27]. However, studies on humans show that there are individual differences in the ability of intestinal flora to produce SFN [28,29].

Meanwhile, studies have reported that in the microbial conversion of glucoraphanin, probiotics, which are a class of active microorganisms that colonize the human intestinal environment, could improve the composition of human intestinal flora and enhance intestinal digestion [30,31]. Probiotics have also shown preventive and therapeutic effects on colorectal cancer, mental disorders, diabetes, and other diseases [32,33]. Therefore, probiotics are widely used in health products, dietary supplements, and prebiotics [34,35,36]. In contrast, studies have shown that lactic acid bacteria (*LAB*) can convert glucoraphanin from broccoli into SFN under suitable conditions, and high SFN concentrations were found in fermented broccoli puree [37,38]. The *LAB* fermentation results also indicated that, in addition to the mammalian gut flora, microorganisms from other sources could also degrade glucoraphanin and produce SFN.

Although microorganisms can convert glucoraphanin into SFN in the gut, the production of SFN is limited due to the insufficient number of microorganisms that can synthesize myrosinase in the intestine [4]. With the discovery of exogenous myrosinase, and the ability of *LAB* to ferment *broccoli* puree to produce high concentrations of SFN, researchers attempted to replicate this process in vivo [37,38].

Microorganisms that can synthesize myrosinase have been isolated from the gut and nature. However, the myrosinase-synthesizing efficiencies of the currently isolated microorganisms are weak, which results in low production rates of SFN. Therefore, it is necessary to modify microorganisms and deliver them into the intestine. This review discusses high myrosinase-synthesizing microorganisms, which were isolated and modified using genetic engineering and artificial selection, and the possibility of delivering these microorganisms into the intestine.

## 2. Intestinal Microorganisms Can Enhance the Utilization of SFN

### 2.1. Structure and Biochemical Characteristics of SFN, Glucoraphanin, and Myrosinase

SFN (1-isothiocyanate-4-(methylsulfonyl) is an isothiocyanates (a general formula R-N=C=S). Due to the presence of an active electrophilic carbon atom in the SFN group (-N=C=S), SFN is easily reversible with thiols under physiological conditions, resulting in pH-sensitive dithiocarbamates reacting with amines and forming thiourea [1,39,40]. This is the reason why SFN is sensitive to temperature and other conditions. SFN is converted by its precursor glucoraphanin under the enzymatic hydrolysis reaction of myrosinase. Meanwhile, glucoraphanin (4-methylsulfinyl butyl glucosinolate) is a methionine-derived aliphatic glucosinolate. Glucoraphanin is widely found in cruciferous plants, especially *broccoli*. Glucoraphanin is a water-soluble sulfur-containing anionic secondary metabolite consisting of a β-glucosinolate N-hydroxysulfate with a side chain center and a β-D-glucopyranose residue [41,42,43]. Glucoraphanin demonstrates no physiological activity. Therefore, the best form of utilization for SFN is to preserve.

Myrosinase, also known as β-glucosidase, is a ubiquitous enzyme in cruciferous plants that can efficiently degrade glucosinolates. The essence of myrosinase is a glycoprotein, and currently found in plants, aphids, and other myrosinase of the glycoside hydrolase family 1 [44,45]. The differences in myrosinase from different sources are mainly reflected in molecular weight, subunit number, and side chain sugar content, which leads to the different ability of myrosinase to degrade glucoraphanin.

### 2.2. Increasing the Intestinal SFN Production Rate Is a Scientific Approach to Enhance SFN Utilization

Although SFN has strong anticancer properties, it is very unstable and loses its biological activity under certain conditions, such as the presence of oxygen, which could reduce the utilization of SFN [46,47,48]. To improve SFN utilization, researchers considered the possibility of directly producing SFN in vivo and conducted in vitro simulation studies [49]. Xu et al. found that glucoraphanin could be converted into SFN under a simulated gastrointestinal environment in vitro, and the maximum conversion rate could reach 46.2%. Moreover, when glucoraphanin was directly fed to germ-free and human-microbiota-associated mice, SFN degradation products were found in the urine of the mice, which indicated that SFN could be produced in the intestinal environment and that SFN could be utilized [50].

Lai et al. used male F344 rats as an animal model and demonstrated that the cecum can also degrade glucoraphanin and produce SFN [27]. Once glucoraphanin was directly gavaged into male F344 rats, SFN was detected in the plasma of the rats after 120 min, and the level of SFN in the plasma remained constant for 1 h. In addition, a study indicated that the intestinal environment not only is a limiting factor for SFN utilization, but also has a promoting effect on SFN utilization [29]. However, humans can consume glucoraphanin-rich *Brassica* vegetables directly instead of consuming some glucoraphanin. Raw *broccoli* is used for studies on humans, and the results indicate that glucosinolates in *broccoli* are degraded in the human body and the degradation products of glucosinolates are detected in the blood and urine of volunteers [51]. Therefore, the studies on humans suggest that SFN can be produced in the human intestinal environment.

Meanwhile, the bioavailability of SFN in raw *broccoli* could reach 37%, which is significantly higher than that in cooked *broccoli*, and the consumption of cooked *broccoli* would delay the absorption of SFN [52]. Egner et al. found that the bioavailability of SFN was far superior to glucoraphanin in the human body [53]. Another study showed that the main reason for the higher bioavailability of SFN than sulforaphane is that glucoraphanin must be hydrolyzed to be absorbed [54].

However, because humans consume more cooked than raw vegetables, researchers have used cooked white *cabbage* to simulate the in vivo degradation of glucoraphanin in a rat duodenal model [55]. The results show that 82% of glucoraphanin is released from white *cabbage* seeds after 10 min, but no degradation of glucoraphanin is detected. However, the in vitro simulation results for the rat duodenum are different from the results from the study on male F344 rats. Therefore, Wu et al. explored the possibility of SFN production by the gut microbiota [28,56]. Research using a male C57BL/6 mouse shows that glucoraphanin could be degraded to SFN in the intestine, and the production of SFN is related to the intestinal flora. Hwang et al. show that 13 pmol/g fresh weight of SFN is produced in the gut after 120 min, and approximately 29% of the SFN is taken up and utilized by cells, indicating that the intestinal environment has a promoting effect on the production and utilization of SFN [57].

Meanwhile, Sangkret et al. found that the main elements affecting SFN production were myrosinase activity, temperature, pH, and reaction time [14]. Recently, researchers obtained a new myrosinase-producing bacterium from marine sediment (Marine Bacterium *Shewanella baltica* Myr-37) [58]. Once the reaction temperature is 40 °C and pH = 7.0, myrosinase can efficiently degrade sulforaphane to SFN in 25 min, the yield is 0.57 mg/mL, and the corresponding SFN conversion efficiency is 89%. However, intestinal myrosinase is also affected by epithiospecifier protein (EP) and sulfur–selenium interaction(S–Se) in the process of SFN formation [48,59,60]. EP interferes with the production of SFN, while S–Se induces the expression of myrosinase gene to produce more myrosinase.

The intestinal environment can degrade glucoraphanin to produce SFN without the action of plant-derived myrosinase, and the main factor for SFN production is the effect of the intestinal flora. However, the mechanisms by which the gut microbiota degrades glucoraphanin to produce SFN, and the gut microbes involved, are not clear. Therefore, researchers investigated the mechanisms of the microbial transformation of glucoraphanin.

### 2.3. Microorganisms Converted Glucoraphanin into SFN Using Myrosinase Synthesis

Myrosinase is a beta-thioglucosidase glucohydrolase that was originally discovered in cruciferous plants; it can resist in vitro damage and degrade glucoraphanin [61,62,63]. Studies have found that some microorganisms can also synthesize myrosinase (Table 1) [64,65,66]. Naoki Tani et al. first isolated a species of *Enterobacter cloacae*, which could synthesize myrosinase, but its molecular weight was smaller than the endogenous myrosinase of the plant. Meanwhile, *Bacteroides thetaiotaomicron* (another dominant species derived from the human colon) could convert glucosinolates into allyl isothiocya-nate [67]. With the further development of research on SFN production by intestinal flora, it was discovered that a variety of intestinal strains can degrade glucoraphanin to produce SFN [68].

**Figure 1:**
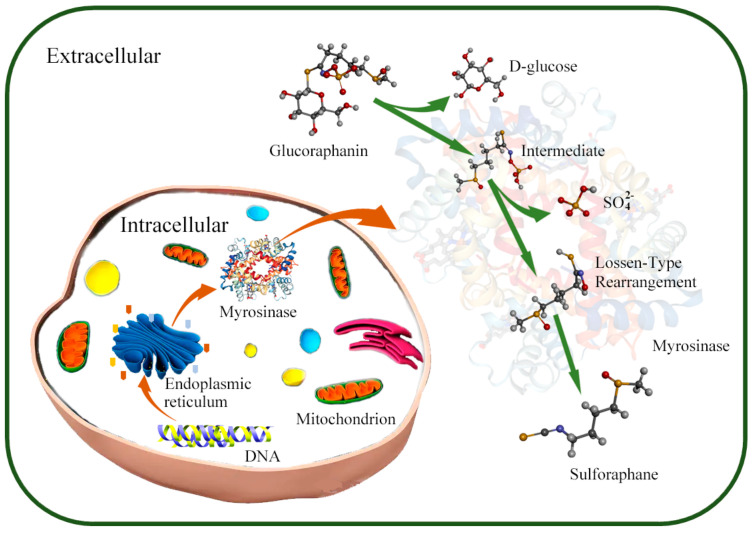
Mechanism of SFN production by microorganisms. In microorganisms, myrosinase is synthesized and secreted to environment through ribosome and endoplasmic reticulum under the myrosinase gene regulation; the glucoraphanin is converted into sulforaphane under the action of myrosinase in extracellular environment.

With the development of research on human intestinal enzyme-producing flora, researchers successfully isolated *Enterococcus gallinarum* HG001 and *Escherichia coli* HG002, which could synthesize myrosinase from the intestines of C57BL/6 mice [69]. However, the mechanisms by which intestinal myrosinase produces SFN remain unclear. Watanabe et al., studying *LAB* as research objects, found that intestinal myrosinase may be involved in the metabolism of glucoraphanin through the β-glucoside-specific IIB, IIC, and IIA phosphotransferase system components (Figure 1) [72]. With the intestinal myrosinase synthesis mechanisms becoming clear, researchers further investigated the myrosinase synthesis capabilities of microorganisms to identify the types of microorganisms that synthesize myrosinase.

Researchers turned to fungal microbes that are ubiquitous. Rakariyatham et al. investigated the ability of *Aspergillus* sp. NR-4201 to synthesize myrosinase [77]. *Aspergillus* sp. NR-4201 converted all glucosinolates into allyl cyanide within 32 h, indicating it can synthesize myrosinase. Subsequently, Nuansri et al. also explored whether *Aspergillus* sp. NR46F13 could synthesize myrosinase [75], and their results show that 3.19 U mL–1 myrosinase was isolated from the medium of this strain after 48 h of culture. With the continuous exploration of microorganisms, researchers isolated *Leclercia adecarboxylata* and *Citrobacter* Wye1, which could synthesize myrosinase, from the soil [73,74].

Palop et al. investigated the potential of 42 *Lactobacillus* species to degrade glucosinolates, and the results indicate that strain R16 shows a strong ability to degrade glucosinolates [78]. In addition, Mullaney et al. explored the different abilities of *L. plantarum* KW30, *Lactococcus lactis subsp. lactis* KF147, and *E. coli Nissle* 1917, while *E. cloacae* was used to degrade glucoraphanin [70]. The comparative research of myrosinase obtained from different microbial sources shows that the myrosinase of the intestinal flora is more capable of degrading glucoraphanin than those of plant-derived microorganisms. Some researchers investigated the feasibility of using *LAB* fermentation as an alternative method to maintain myrosinase activity and enhance the bioconversion of glucosides into SFN [37]. The fermentation results show that the fermentation of *LAB* could achieve stable SFN production.

Some researchers further explored the effect of high-temperature sterilization or preheating *broccoli* puree on the fermentation of *LAB* to produce SFN [38,71]. The results show that high-temperature sterilization and preheating *broccoli* puree enhances the yields of SFN produced by *LAB* fermentation, and the SFN yield from *broccoli* puree that was preheated in advance is 16 times higher than that of non-preheated *broccoli* puree. Xu et al. show that using *Pediococcus pentosaceus* for fermentation in *broccoli* juice also produces more SFN [76]. Exogenous myrosinase could stably produce SFN under the condition of sufficient raw materials, which provides a new route for SFN production. In addition, the results indicate that microbial-derived myrosinase has the same efficacy as plant-derived myrosinase, but microbial-derived myrosinase is easier to obtain in vitro.

Microorganisms that could synthesize myrosinase have also been isolated from soil and *broccoli*, in addition to those isolated from the intestinal environment. However, the myrosinase-synthesizing abilities of the microorganisms were restricted by the culture conditions and their gene sequences; therefore, it was necessary to further investigate the factors that restrict the synthesis of high-yield myrosinase enzymes.

## 3. Improving the Myrosinase-Synthesizing Abilities of Microorganisms Is a Critical Approach to Increase SFN Production

### 3.1. Culture Conditions Influence the Efficiency of Myrosinase-Synthesizing Microorganisms

**Figure 2:**
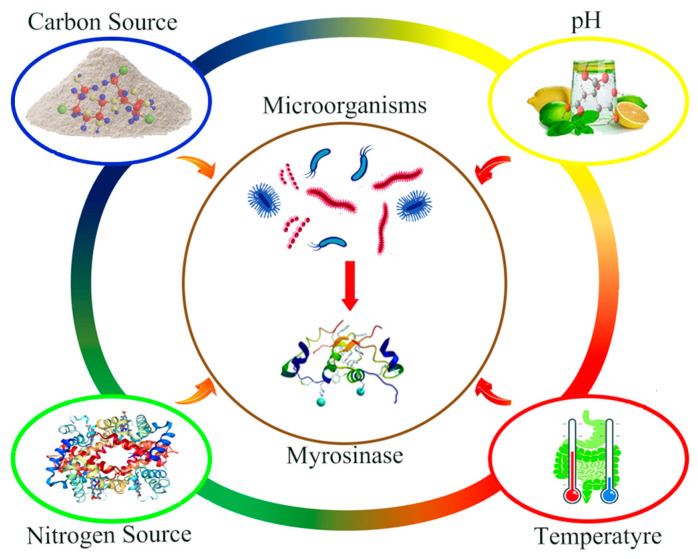
Factors affecting the synthesis of myrosinase by microorganisms.

Microorganisms are organisms that can adapt to various complex growth environments and exhibit different physiological characteristics and life activities [79,80]. Enzymes synthesized by microorganisms have a long history of application in the fermentation industry, such as in the production of cheese, yogurt, milk powder, and the Chinese liquor, Daqu. Research shows that the efficiency of microbial myrosinase production is affected by the culture conditions (Figure 2) [79,81,82]. In these studies, nutrient content is an important factor in determining the synthesis of myrosinase by microorganisms, and the external environment also affects the myrosinase production efficiency of microorganisms. Therefore, it is very important to determine the factors that affect the myrosinase-synthesizing activities of microorganisms that stably express myrosinase genes.

Nutrients are important raw materials for the growth and metabolic activities of microorganisms, because nutrients can affect the ability of microorganisms to produce myrosinases, which can, in turn, affect the production of target substances [83]. A study reported that applying inulin as a carbon source can promote the growth of *Lactobacillus* and decrease enzymatic activity [79]. Lactose and galactose are mandatory carbon sources for promoting methionine gamma-lyase production by *L. plantarum*, and the results show that the level of methionine gamma-lyase production by *L. plantarum* with galactose as the carbon source increases by 16.7% [84]. Microorganisms have diverse preferences for their carbon sources; for example, L. brevis 145 prefers fructose [85]. However, *LAB* uses glucose to synthesize myrosinase [38].

Amino acids are another type of nutrient that can limit microbial myrosinase production, and L-methionine is an important limiting nitrogen source for methionine gamma-lyase synthesis; therefore, nitrogen sources can affect the microbial synthesis of myrosinase [84]. Studies on the synthesis of fibrinolytic protease in *Bacillus flexus* BF12 show that the best nitrogen source is beef extract [86]. West et al. demonstrate that uracil or cytosine is an important nitrogen source for the production of the polysaccharide curdlan by *Agrobacterium species*, and the polysaccharide concentration increases by 1.7-fold after adding pyrimidine [87]. In addition, a study on fermenting *broccoli* juice with *Pediococcus pentosaceus* shows that a nitrogen source containing large amounts of methionine is required as a supplement during the conversion of glucoraphanin into SFN by *P. pentosaceus* [76].

Although nutrients have important effects on the synthesis of myrosinase by microorganisms, environmental factors can also not be ignored [88,89,90]. A study on the abilities of microorganisms to produce pectinase at various pH and temperature ranges shows that the optimum pH for *B. sphaericus* MFW7 is 6.5 and the optimum temperature for *Brevibacillus borstelensis* (P35) is 60 °C [91]. However, environmental factors such as Tween-80, the initial moisture ratio, and magnesium sulphate all have effects on the production of *Bacillus* MSK-01 by laccase [92]. Moreover, a study reports that pH and temperature also affects the yield of myrosinase by *Citrobacter* Wye1, and the optimum pH and temperature are found to be 6.0 and 25 °C, respectively [73].

Carbon and nitrogen sources are the main nutrients that limit myrosinase synthesis by high-yielding microorganisms, and different microorganisms show diverse requirements for their carbon and nitrogen sources. Culture environment is another factor that limits high myrosinase synthesis, especially pH and temperature. Therefore, the optimal myrosinase-producing culture conditions for different high-myrosinase-synthesis microorganisms vary, which also provides a reference for the in vivo nutrient intake before the in vivo transport of high-myrosinase-synthesis microorganisms.

### 3.2. Modified Microorganisms with Higher Myrosinase-Synthesizing Abilities Can Promote SFN Production

Researchers have isolated microorganisms with myrosinase-synthesizing abilities from the gut flora and nature, but the myrosinase synthesis rates are not high, which results in low SFN utilization [69,93]. With the further development of gene research, targeted gene modification has become the most commonly used method for culturing microorganisms with high myrosinase yields. Genetically engineered myrosinase microorganisms with high-enzyme-producing capacities can be rapidly used in production [35].

Currently, some microorganisms that can produce exogenous myrosinase were discovered in the gastrointestinal tract and nature. However, the ability of these microorganisms to synthesize myrosinase is weak [65,69,70]. Research shows that transformation technology is the best approach to increase myrosinase production [94]. The principle of transformation is changing the permeability of the bacterial cell membrane, and the plasmid containing the target gene is taken into the bacterial cell [95]. Thus, two main factors restrict the success of bacterial transformation: the first factor is the generation of bacterial competent cells, and the second factor is that the plasmid carrying the target gene can correctly express the target gene in bacterial cells [96].

The technology for preparing competent cells is mainly divided into chemical, electrical, and physical methods [95]. In general, it is used to stimulate the microbial cell membrane by changing the external environment to obtain a microbial cell state that can easily take in external DNA [97]. In addition, the transformation effects of using arginine–glucose-functionalized hydroxyapatite nanoparticles to directly introduce the recombinant plasmid into *Staphylococcus aureus* MTCC 737 and *E. coli* DH5α are 10^9^ CFU/µg plasmid DNA and 10^7^ CFU/µg plasmid DNA [98]. The genes transformed by *S. aureus* MTCC 737 have extremely high stability, which is of great significance for the construction of recombinant microorganisms transformed with high SFN. Moreover, Pandey et al. used β-alanine/citric acid as a DNA carrier to deliver exogenous DNA up to 10 kb (kilobase, a common unit of DNA length, is equal to 1000 bp) to *E. coli* [99]. Pandey et al. show that the plant myrosinase gene can be introduced into microorganisms through nanoparticles, which will further improve the culture efficiency of microorganisms with high SFN transformation.

However, the stable expression of recombinant genes is another important factor that restricts the effect of bacterial transformation. Currently, the most commonly used recombinant gene vector is a plasmid, and the target protein could be successfully obtained after the target gene was transferred into *E. coli* [100]. Jain et al. found that RarA protein can stabilize the RecA-independent recombination of *E. coli*, especially on 200 bp homologous gene recombination [101]. The discovery of the RarA protein proves that some endogenous proteins of *E. coli* have the function of stabilizing recombinant genes. A two-plasmid-based pIT5 (IT = integrating, terminator protected) is being used for the transformation of *E. coli* [102]. The gene transformation process of pIT5 (containing a helper plasmid and a specialized recombinant plasmid) can be repeated multiple times to obtain more transformed strains.

The novel IncI1-type conjugative helper plasmid pSa42-91k could fuse with the plasmid pBackZeroT loaded with the MGEs ISEcp1 and IS15DI genes, resulting in gene transfer [103]. The discovery of plasmid pSa42-91k expanded the vector of gene transformation, reduced the secondary shear modification of the target gene, and reduced the risk of gene leakage. *E. coli* DH5α successfully expressed the transformed hematone gene and green fluorescent protein gene and obtained the target protein [104]. Therefore, Cabeci et al. transferred the myrosinase gene of *Citrobacter* Wye1 into *E. coli* [73]. The exogenous myrosinase gene could be expressed normally in *E. coli*, and the myrosinase synthesized by *E. coli* showed strong specificity for glucoraphanin. Wang et al. found that the myrosinase Rmyr gene from *Rahnella inusitata* could also be expressed heterologously in *E. coli* BL21 (DE3) [105]. The myrosinase synthesized by DE3 could degrade 97.84% of glucoraphanin. These studies suggest that genetically modified microorganisms synthesize more myrosinase to produce SFN.

However, the myrosinase gene in *broccoli* could also be expressed heterologously in microorganisms [106]. Both the myrosinase synthesized by genetically modified *Saccharomyces cerevisiae* and *E. coli* has higher catalytic efficiencies, but the activity of the myrosinase synthesized by *S. cerevisiae* is four times higher than that produced by *E. coli*. Recombinant *Yarrowia lipolytica* successfully expresses the myrosinase gene from *Arabidopsis thaliana*, and the yield of SFN increases by 92.53% after the recombinant *Y. lipolytica* is fermented 10 times [107]. Rosenbergova et al. achieve heterologous expression of the *A. thaliana* myrosinase gene in *Pichia pastoris*, and the myrosinase synthesized exhibits high activity [108]. Rosenbergova et al. found that microorganisms can be genetically engineered to have high levels of myrosinase synthesis.

At present, researchers found that there are three main sources of myrosinase genes, namely, plant myrosinase gene, microbial myrosinase gene, and animal myrosinase gene [44,45,73]. Among them, plant myrosinase genes and microbial myrosinase genes are the most commonly used heterologously expressed myrosinase genes, such as *Arabidopsis* genes THOGLUCOSIDASE1 (TGG1) and THIOGLUCOSIDASE2 (TGG2) [109,110].

Although targeted modifications of microorganisms by genetic engineering could achieve microbial metabolic factories with high levels of myrosinase synthesis, genetically engineered microorganisms may have the risk of gene leakage and contamination of the host genes in the human intestinal environment [43,111]. Therefore, it is safer and more reliable to obtain strains with high myrosinase synthesis through traditional artificial selection methods. Currently, researchers have isolated some bacterial samples with myrosinase genes that can be stably expressed, and bacterial myrosinase can stably convert glucoraphanin into SFN [69,72,74]. By configuring different types of deficient media, researchers conducted screening and optimization studies of microorganisms and obtained microorganisms that can stably express high-yield myrosinases through morphological identification, biochemical identification, and 16S rDNA sequence analysis [112].

Microorganisms with high myrosinase synthesis can be obtained through genetic-engineering-directed modifications and manual screening of defective media. The abilities of the screened microorganisms, which are high myrosinase-yielding, to convert glucoraphanin into SFN are significantly enhanced. However, the efficiency of these microorganisms in degrading glucoraphanin to produce SFN is still low. The main limiting factor is the low content of microorganisms, thereby necessitating the intake of microorganisms with a protective delivery system.

## 4. A Microencapsulated Delivery System Can Protect Microorganisms into Intestines

Although the human gut flora is confirmed to convert glucoraphanin into SFN, the ability of the intestinal flora to produce SFN is low due to limitations in the quantity and ability to synthesize myrosinase. As probiotic or intestinal strains that can produce SFN have been successfully isolated [69,72], transporting microorganisms with high myrosinase synthesis levels into the intestine has become an important approach to improve the ability of the intestinal flora to produce SFN. As the gut environment is complex, microorganisms delivered into the body that have high myrosinase synthesis are easily attacked by gastric acid and bile, which can reduce their survival rates [113]. Encapsulation, coating, and embedding technologies have been applied to the in vivo delivery of microorganisms to cope with the complex human environment and to improve the survival rates and stability of microorganisms (Table 2).

**Figure 3:**
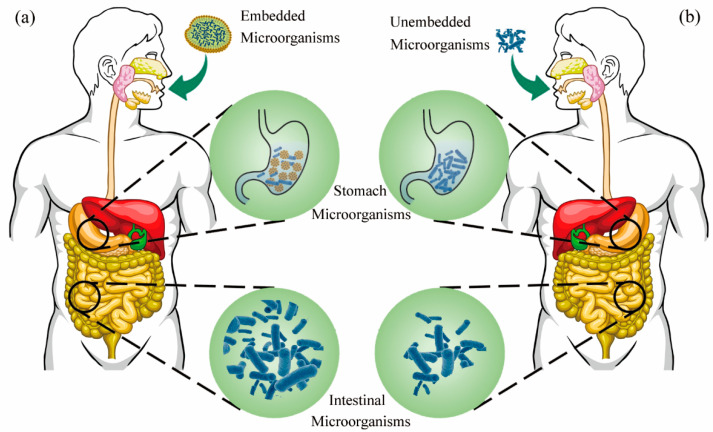
Protect effect of micro-encapsulated delivery system on probiotics. (**a**) Microorganisms enter the intestine under the protection of microcapsules; (**b**) microorganisms enter the intestine without the protection of microcapsules.

The current route for the in vivo delivery of microorganisms is by oral administration, and encapsulated probiotic supplements can stably exist in the body and colonize the intestinal tract and have certain abilities to clear intestinal bacteria (Figure 3) [116]. Microencapsulation or nanoencapsulation is the most commonly used technology to encapsulate microorganisms, and encapsulated microorganisms with different effects can be obtained by changing the encapsulation materials [36]. To resist the invasion of the acidic environment, microencapsulated technology was researched to improve the stability of encapsulated microorganisms and promote the ability of intestinal flora to metabolize glucoraphanin to produce SFN.

**Figure 4:**
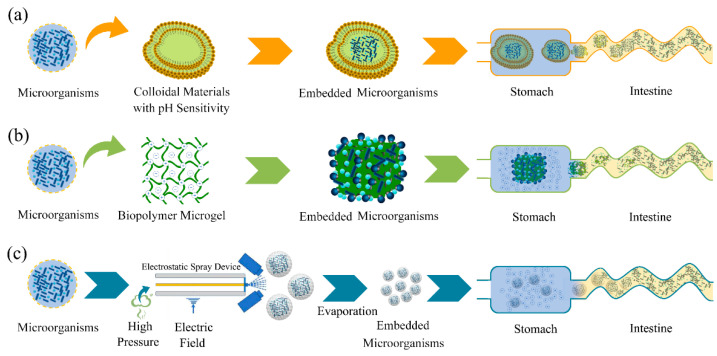
The protection principle of different embedding methods for microorganisms. (**a**) Microspheres/microcapsules mainly used colloidal materials with pH sensitivity to achieve multilayer embedding of microorganisms, and microcapsules disintegrated layer by layer to help microorganisms targeting release at the specific site in intestine. (**b**) Biopolymer microgels mainly protected microorganisms by adding colloidal antacids. Colloidal antacids maintained a neutral internal pH of biopolymer microgels under stomach conditions due to the ability of the hydroxyl ions for colloidal antacids to neutralize hydrogen ions arising from the gastric fluids. (**c**) Electrospraying made the emulsion dispersed into uniform and stable micron or nanometer droplets by high pressure and applied electric field.

Furthermore, a modified alginate material was researched through material modification, and hydrogel beads were created from the modified alginate material [117]. The microorganisms protected by the hydrogel beads not only were protected from the gastric environment, but could also be released at different intestinal sites by controlling the content of the modified alginate. Cheng et al. used alginate and protamine as embedding materials and constructed an enzyme-triggered fuse-like microcapsule using combined electrostatic droplets [118]. The multilayered microcapsules have good protection against *E. coli* MG1655 during oral administration, and almost no *E. coli* is released in the gastric environment, but these bacteria are released 1 h after entering the intestinal environment (Figure 4a).

However, the oral core–shell microspheres constructed with sodium alginate and calcium ions as package materials exhibit colon-targeting properties. In addition, the residence times of local microorganisms are prolonged for the collapse release mechanism, and the numbers of microorganisms increases the bioavailability [119]. Kamguyan et al. developed a chitosan-coated microcontainer using *L. rhamnosus* as a model strain [120]. The chitosan-coated microcontainer was stable in the gastrointestinal environment and could specifically transport *L. rhamnosus* to the cecum or colon. The microorganisms were encapsulated in chitosan-coated alginate microparticles using emulsion technology, and feeding experiments show that the alginate microparticles can be used as a controlled release system to deliver live microorganisms to the gastrointestinal tract of abalone [121].

Biopolymer microgels are another form of encapsulation that can stabilize the entrapped microorganisms within the gastrointestinal environment and release them at the target intestinal site (Figure 4b) [122]. The stability of the microgels in the study was dependent on the different additives: acid-resistant microgels > control microgels > acid-resistant nanoemulsion microgels. Simulated in vitro colon experiments show that microorganisms are released due to fermentation of the microgels in the colon. Thiolated oxidized konjac glucomannan microspheres could achieve stable colonization of *bifidobacteria* within the intestinal environment [123]. Thiolated oxidized konjac glucomannan microspheres could also balance the intestinal flora and increase the abundance of *bifidobacteria*. Some researchers suggest that the multilayer electrospun structure is an important development direction for probiotic encapsulation and has important commercial value [124].

Electrospraying is a technique similar to electrospinning (Figure 4c) [125]. Small and uniform microcapsules can be prepared using electrospraying technology, and the activity and entrapment rate of electrospraying substances are higher than those of traditional spray drying methods. Electrospraying results of Premjit and Mitra show that 92.93% of *Leuconostoc lactis* is encapsulated, and the activity loss of encapsulated *Leuconostoc lactis* is 0.663 log CFU/g [126]. Huang et al. show that the encapsulation rate of electrospraying for *L. plantarum* is as high as 92.53%, and the encapsulation activity of *L. plantarum* by electrospraying is significantly higher than that by spray drying [127]. This also shows that electrospray technology has the characteristics of high encapsulation efficiency and high microbial activity, and also provides a reference for the encapsulation of SFN-transformed microorganisms.

Microencapsulation is another important form of microorganism encapsulation, and it has achieved long-term development [114,115,128]. Maillard conjugates and nanomaterials are currently one of the most commonly used microbial microencapsulation materials, which have the advantage of maintaining microbial stability and improving microbial intestinal colonization [129,130]. An in vitro simulation experiment shows that microcapsules can also improve the ability of microorganisms to produce myrosinases. Microencapsulation can deliver microorganisms that can synthesize myrosinase into the human intestinal environment, promote the stable existence and colonization of microorganisms in the intestinal environment, and can increase myrosinase synthesis.

## 5. Conclusions

Although intestinal microorganisms can synthesize myrosinase to degrade glucoraphanin and produce SFN, the myrosinase synthesis ability and colonization of the gut microbes are limited. Enhancing the myrosinase synthesis ability of gut microbes is the main effective means to promote the utilization rate of SFN. The application of transformation and microencapsulation technology to improve the myrosinase synthesis and colonization ability is feasible, and these technologies can enhance the absorption and utilization of SFN in the intestinal tract. Therefore, industries that produce high-yielding myrosinase microorganisms should consider transformation and microencapsulation technology, and it is important to provide technical support for the production and application of SFN. Meanwhile, attention should also be paid to the risk of leakage of transformed microbial genes and the uncontrollability of establishing a high-yield myrosinase microbial system in the intestine.

## Figures and Tables

**Table 1:** Microorganisms with myrosinase synthesis function.

Strain	Source	Substrate	Products	Transformation Ability	References
*Lactobacillus agilis* R16	NS	Glucoiberin/glucoraphanin	NS	10%	[68]
*Enterococcus casseliflavus* CP1	Human feces	Glucoiberin/glucoraphanin	Iberin/SFN	40–50%	
*Escherichia coli* VL8	Human feces	Glucoiberin/glucoraphanin	Glucoiberverin/glucoerucin	80–90%	
*Enterococcus gallinarum* HG001	Mouse feces	Glucosinolate	Isothiocyanate	39.54%	[69]
*Escherichia coli* HG002	Mouse feces	Glucosinolate	Isothiocyanate	29.17%	
*L*. *plantarum* KW30	NS	Glucoraphanin, etc.	SFN, etc.	30–33%	[70]
*Lactococcus lactis subsp. lactis* KF147	NS	Glucoraphanin, etc.	SFN, etc.	30–33%	
*E. coli Nissle* 1917	NS	Glucoraphanin, etc.	Glucoerucin, etc.	65–78%	
*Bacteroides thetaiotaomicron*	Human feces	Sinigrin	Allyl isothiocyanate	NS	[71]
*Companilactobacillus farciminis* KB1089	Pickles	Sinigrin	Allyl isothiocyanate	NS	[72]
*Citrobacter* Wye1	Soil	Sinigrin	Allylcyanide	NS	[73]
*Leclercia adecarboxylata*	Soil	Sinigrin	Allylcyanide	NS	[74]
*Aspergillus* sp. NR46F13	Soil	Sinigrin		NS	[75]
*LAB*	*Broccoli*	Glucoraphanin	SFN	NS	[37]
*LAB*	*Broccoli*	Glucoraphanin	SFN	NS	[38]
*Pediococcus pentosaceus*	Natural fermented *cherry* juice	Glucoraphanin	SFN	NS	[76]
*Aspergillus* sp. NR-4201	NS	Glucosinolate	Allylcyanide	NS	[77]
*Lactobacillus agilis* R16	NS	Sinigrin	Allyl isothiocyanate	NS	[70]

Note: Not explicitly stated (NS).

**Table 2:** Delivery system of microorganisms.

Bacterial Strains	Compositions	Delivery Systems	Functions	References
*Lactobaccillus casei* NCDC 298	Modified alginate	Hydrogel	Protects probiotics from enzymatic hydrolysis	[106]
*E. coli* MG1655	Alginate and protamine	Microcapsule	Protects probiotics from acidity and bile salts	[107]
NS	Alginate	Microsphere	Antiacid and colon targeting	[108]
*L. rhamnosus*	Chitosan	Microcontainers	Targeted delivery of probiotics	[109]
*Exiguobacterium*	Chitosan and alginate	Microparticles	Targeted delivery of probiotics	[110]
*Bifidobacterium pseudocatenulatum*	Calcium alginate	Microgels	Improve the stability of probiotics	[43]
*Bifidobacterium*	Thiolated oxidized konjac glucomannan	Microspheres	Improves intestinal colonization of probiotics	[111]
*Lactobacillus rhamnosus* GG	Amylopectin	Nanofibers	Enhanced probiotic delivery capabilities	[112]
*Bifidobacterium*	Alginate	Microcapsules	Improve the survival rate of probiotics	[114]
*Streptomyces lividans* 66	Alginate	Micro-encapsulation	Improve the enzyme production capacity of probiotics	[115]

Note: Not explicitly stated (NS).

## Data Availability

Not applicable.
